# Identification of a Novel Cuproptosis-Related Gene Signature in Eutopic Endometrium of Women with Endometriosis

**DOI:** 10.1007/s43032-022-01130-7

**Published:** 2022-12-06

**Authors:** Jiahui Wei, Baoyi Huang, Yingqi Nong, Qianyu Zhang, Wenjuan Liu, Yanni Xie, Tong Peng, Wei Wang, Xiangping Liang, Qiuyun Li, Fenghua Liu

**Affiliations:** 1 Guangzhou Medical University, Guangzhou, 511495 China; 2 Department of Reproductive Health and Infertility, Guangdong Women and Children Hospital, Guangzhou, 511442 China

**Keywords:** Endometriosis, Cuproptosis, Eutopic endometrium, Bioinformatics analysis, *PHDA1*

## Abstract

Endometriosis (EMs) is a life-long endocrine disorder and a common cause for female infertility and pelvic pain. The key characteristics of eutopic endometrium of EMs patients are high proliferative and migratory potentials. Cuproptosis is a recently identified copper- and-mitochondrial-dependent regulated cell death. Regretfully, its role in EMs remains unclear. In this study, Kyoto Encyclopedia of Genes and Genomes analyses of differentially expressed genes (DEGs) indicated strong activation of the PI3K-Akt-mTOR pathway and biological process analysis reported positive regulation of kinase activity. Next, we screened 11 cuproptosis-related DEGs and found all of them were downregulated in the EMs group, which indicated the suppression of cuproptosis in EMs. One key cuproptosis-related gene, *PDHA1*, was selected via support vector machine, random forest algorithm and lasso regularization to build a risk-scoring model, which was tested in both internal and external validations. In conclusion, the downregulation and kinase activity of *PDHA1* may function with the PI3K-Akt-mTOR pathway in some way, which could suppress the cuproptosis level and account for the cancer-like pathology in EMs.

**Supplementary Information:**

The online version contains supplementary material available at 10.1007/s43032-022-01130-7.

## Introduction

Endometriosis (EMs), first described by Shroen in 1690 [1], is a heterogeneous disease defined by the appearance of endometrium-like lesions outside the uterine cavity [2]. Statistically, up to 35–50% of women with pelvic pain or infertility have suffered from this disease [3]. In 1927, Sampson [4] proposed the most common and well-accepted etiological theory of EMs: retrograde menstruation initiates the endometrial tissue’s transplantation outside the uterine cavity. According to this classic theory, the origin of ectopic lesions of EMs is eutopic endometrium. Nevertheless, while retrograde menstruation occurs in approximately 90% of women of reproductive age, only slight friction develops EMs [5]. To advance Sampson’s theory, many researchers have suggested that compared with its matched ectopic lesions, eutopic endometrium from women with EMs displays different molecular and functional features, including transcriptome features [6, 7], steroid hormone signaling [8] and cell death mechanism [9, 10]. Thus, there is a need for specific studies oriented towards the eutopic endometrium of EMs.

A recent study published in *Science* proposed an exclusive type of programmed cell death (PCD) termed cuproptosis, which is triggered by intracellular copper accumulation [11]. Overwhelming copper would directly bind to lipoylated proteins, resulting in oligomerization and a toxic gain of function. More importantly, all these proteins are located in mitochondria and involve in mitochondrial respiration, especially the tricarboxylic acid (TCA) cycle. Several studies have reported impaired copper ionic homeostasis in EMs. Turgut et al. [12] compared serum copper and ceruloplasmin levels between women with and without EMs and found a significant elevation in EMs groups, which may be closely related to increased oxidative stress levels. Likewise, another research found that urinary copper is relatively higher in patients than in disease-free women [13]. Besides, mitochondrial metabolism disorders [14] and DNA mutation [15] have also been reported in EMs.

Thus, our study is aimed at preliminarily exploring the cuproptosis-related molecular and functional alterations in the eutopic endometrium, which might lay a foundation for the future investigation of the cuproptosis-targeting therapy in EMs.

## Materials and Methods

### Data Downloading and Preprocessing

**Fig. 1:**
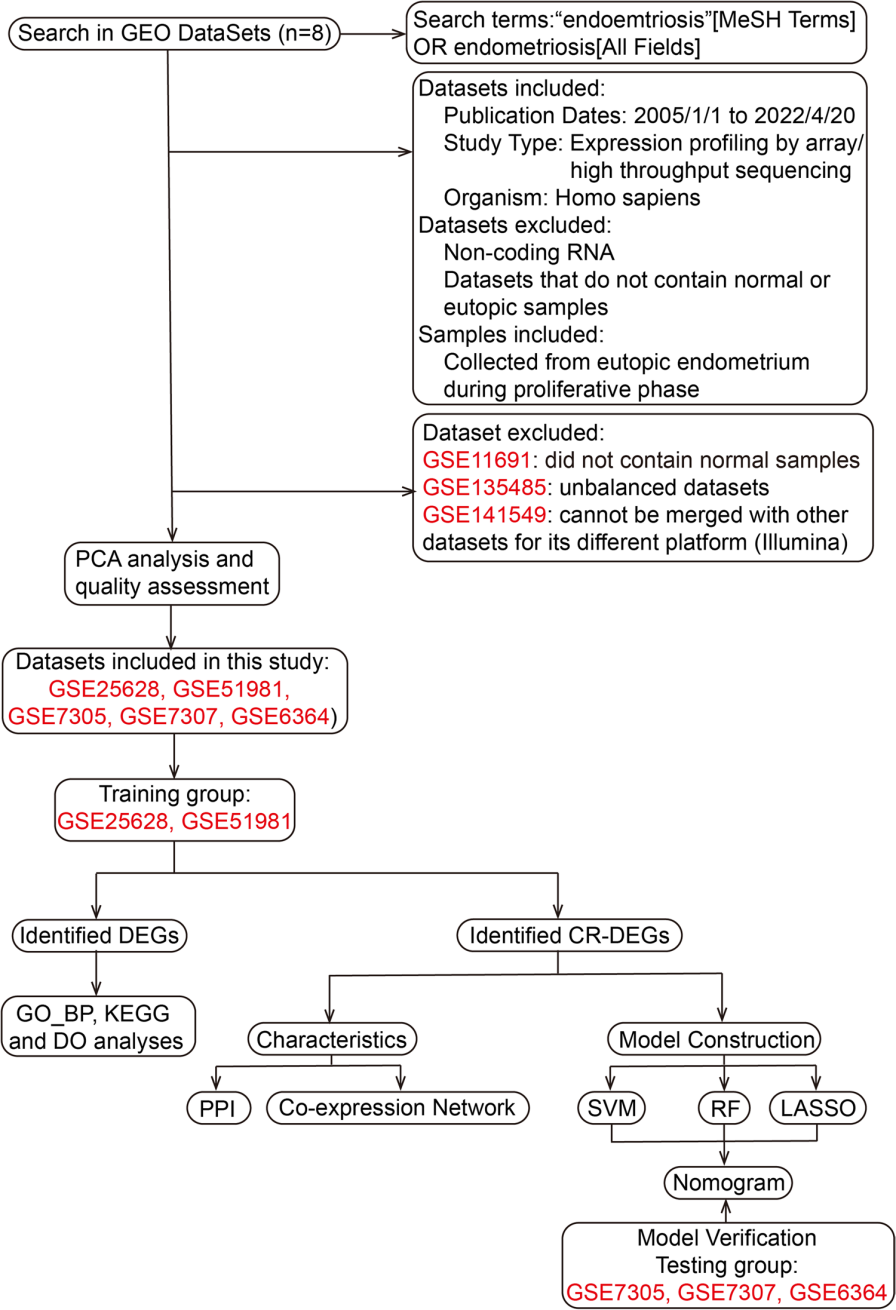
The flowchart of the whole study. GEO Gene Expression Omnibus, PCA principal component analysis, DEGs differentially expressed genes, CR-DEGs cuproptosis-related DEGs, BP_GO biological process analysis of Gene Ontology, KEGG Kyoto Encyclopedia of Genes and Genomes, DO Disease Ontology, PPI protein–protein interaction, SVM support vector machines, RF random forest algorithm, LASSO logistic regression with lasso regularization

First, GSE141549, GSE135485, GSE51981, GSE25628, GSE11691, GSE7305, GSE7307 and GSE6364 were downloaded as candidate datasets from the GEO database (https://www.ncbi.nlm.nih.gov/geo/). The basic information of retrieved datasets is shown in Table 1. The candidate datasets were preliminary filter based on the criteria shown in Fig. 1. Second, after using the R package ‘LIMMA’ to normalize candidate datasets, the principal component analysis (PCA) was conducted to verify their quality [21]. Datasets with poor performance in PCA were excluded from further analyses. Third, these datasets were merged into a training dataset and a testing dataset, respectively, whose batch effect was removed via R package ‘sva’ [22]. R software (version 4.1.3) and R Studio (version 1.0.143) were used to run all R packages and perform all analyses in this study.

**Table 1:** The basic information of retrieved datasets of EMs

Study type	Accession ID	Platforms	NM samples	EU samples	References
Array	GSE141549	GPL10558 Illumina HumanHT-12 V4.0 expression beadchip, GPL13376 Illumina HumanWG-6 v2.0 expression beadchip	7	17	Gabriel et al.[16]
High-throughput sequencing	GSE135485	GPL21290 Illumina HiSeq 3000	4	54	Unpublished
Array	GSE51981	GPL570 [HG-U133_Plus_2]	38	26	Tamaresis et al. [17]
Array	GSE25628	GPL571 [HG-U133A_2]	6	9	Crispi et al.[7]
Array	GSE11691	GPL96 [HG-U133A]	-	9	Hull et al.[18]
Array	GSE7305	GPL570 [HG-U133_Plus_2]	8	8	Hever et al.[19]
Array	GSE7307	GPL570 [HG-U133_Plus_2]	23	19	Unpublished
Array	GSE6364	GPL570 [HG-U133_Plus_2]	5	6	Burney et al.[20]

### Identification of Differentially Expressed Genes and Cuproptosis-Related Differentially Expressed Genes

The expression profiles of normal endometrium (NM) and samples from eutopic endometrium of patients (EU) were compared to screen the differentially expressed genes (DEG) via Student’s *t*-test. The threshold criteria were log(fold change) > 0.5 and adjusted *p* value < 0.05. Then, a volcano plot was drawn via R package ‘LIMMA’ to display these DEGs [21].

Cuproptosis-related genes were extracted from the study published by Tsvetkov et al. [11]. The intersection of obtained cuproptosis-related genes and DEGs yielded a group of cuproptosis-related DEGs (CR-DEGs). The heat map and boxplot of CR-DEGs were performed with R packages ‘LIMMA’, ‘pheatmap’, and ‘ggpubr’ [21, 23, 24].

### Functional Enrichment Analysis of DEG

The DEGs were mapped to biological process (BP) terms (http://www.geneontology.org/), Kyoto Encyclopedia of Genes and Genomes (KEGG) terms (https://www.genome.jp/kegg/) and Disease Ontology (DO) terms (http://disease-ontology.org/), respectively. The criterion for statistical significance was adjusted *p* value > 0.05. The enrichment analyses were all performed with R packages ‘org.Hs.eg.db’, ‘clusterProfiler’ and ‘enrichplot’ [25, 26].

### Protein–Protein Interaction and Co-expression Network of CR-DEGs

The protein–protein interaction (PPI) network of the CR-DEGs was generated by the online database STRING (https://cn.string-db.org/) with the species limited to ‘*Homo sapiens*’ and a confidence score > 0.7. The co-expression network was performed using R packages ‘igraph’ [27].

### Screening Characteristic CR-DEGs

Three machine learning approaches were applied to screen characteristic CR-DEGs, including support vector machine (SVM), random forest (RF) algorithm and logistic regression with lasso regularization. SVM was performed using R packages ‘e1071’ with linear kernel [28]. Tenfold cross-validation was conducted to assess accuracy of the SVM classifiers, which split the dataset into ten random subsets [29]. Each partition was taken as a testing set and the remainder as the training set. RF analysis was performed through R packages ‘randomForest’ [30]. The number of decision trees was adjusted in terms of the out-of-bag error rate. The importance of each variable was evaluated by mean decrease of the Gini index (MDG); only genes with MDG scores larger than four would be collected for further analysis. Lasso regularization was performed to avoid overfitting via R package ‘glmnet’ [31]. Finally, the intersection of obtained genes from these three algorithms was created by a Venn diagram (http://bioinformatics.psb.ugent.be/webtools/Venn/).

### Construction and Validation of a Nomogram

Next, a nomogram for risk assessment was constructed using the binomial logistic regression model based on the output characteristic CR-DEGs. Calibration grams, concordance index (*C*-index) and receiver operating characteristic (ROC) were utilized to evaluate the quality of the risk-scoring model. Following this, the testing group was used to verify the quality of the model externally. These steps were achieved via ‘dplyr’ [32], ‘timeROC’ [33], ‘rms’ [34] and ‘ROCR’ [35].

## Result

### Data Selecting and Preprocessing

**Fig. 2:**
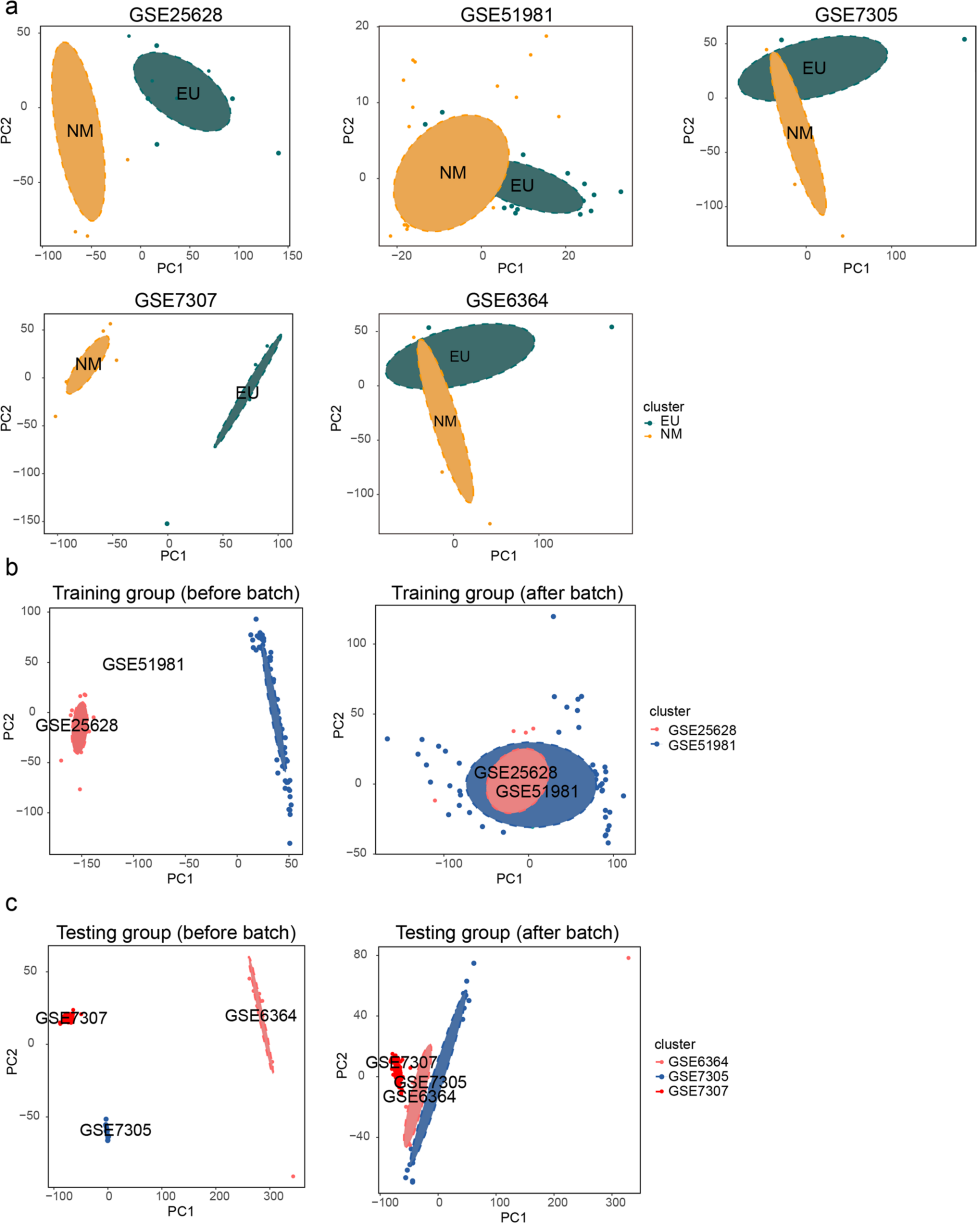
The quality assessment of datasets. **a** The PCA showing quality of each selected dataset. **b**, **c** The PCA before and after batch effect

After eliminating samples collected during the non-proliferative phase, three datasets were excluded from this study: GSE11691 did not contain normal samples; GSE135485 displayed unbalance between NM samples and EU samples (Table 1), while GSE141549 was measured by an Illumina expression beadchip, which made it difficult to merge with other datasets run on Affymetrix human chip. Next, PCA showed that EU samples could be well-distinguished from NM samples in each selected dataset (Fig. 2a). Thus, GSE51981 and GSE25628 were reserved as the training group (44 NM samples and 35 EU samples); GSE7305, GSE7307 and GSE6364 were reserved as the testing group (36 NM samples and 33 EU samples). The disease stage of involved EU samples is displayed in Supplementary Table 1 according to the revised American Fertility Society (rAFS) classification [36]. Next, PCA indicated that batch effect was well-removed from both the training dataset and the testing dataset (Fig. 2b c).

### Identification and Functional Analyses of DEGs

**Fig. 3:**
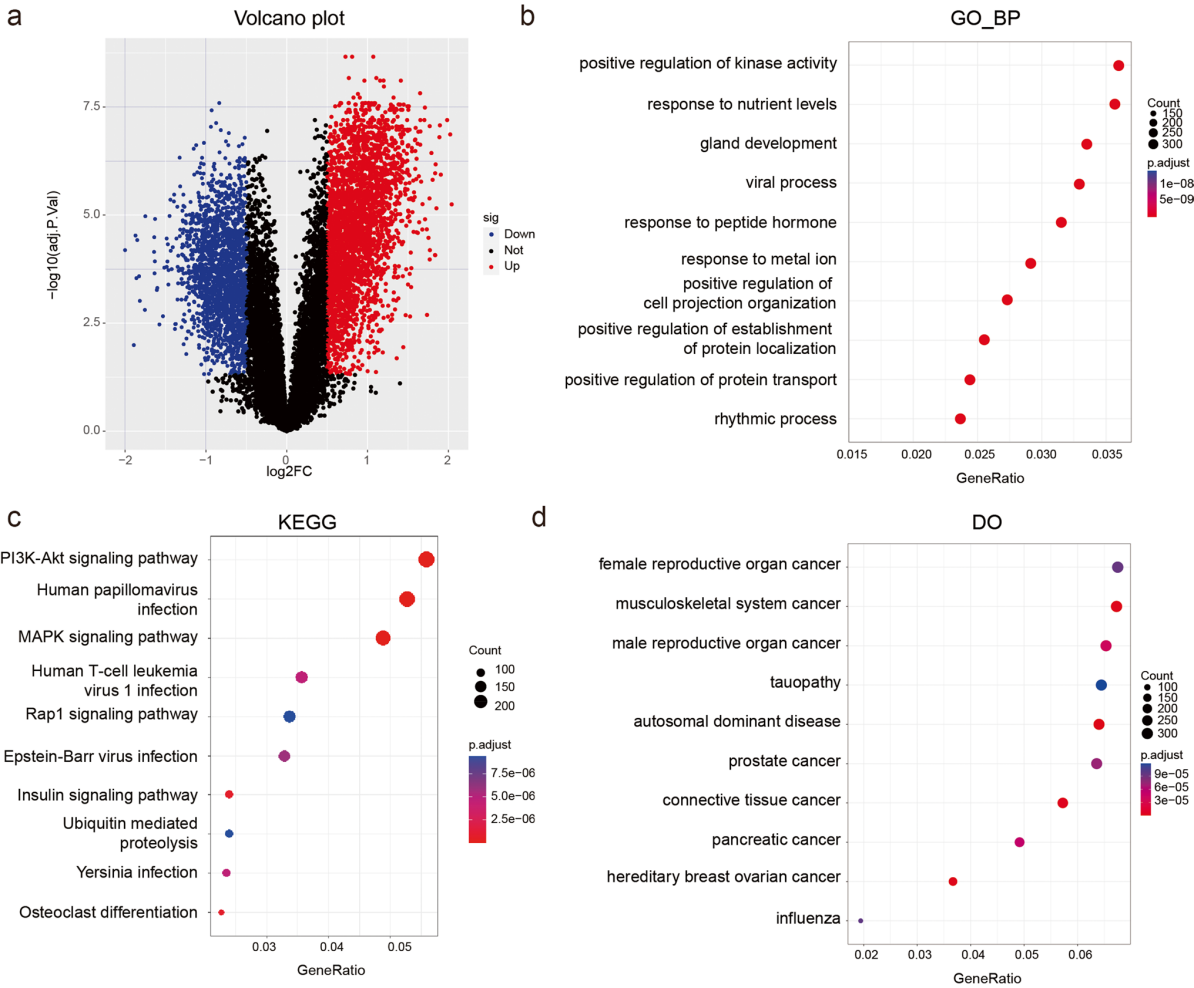
Identification and characteristics of DEGs. **a** The volcano plot showing DEGs (red points, relatively upregulated genes; blue points, downregulated genes). **b** Top 10 BP terms. **c** Top 10 pathways in KEGG analysis. **d** Top 10 related diseases in DO enrichment analysis. Color intensity denoted the level of DEGs’ enrichment in each functional analysis

In total, 5022 DEGs (log(fold change) > 0.5 and adjusted *p* value < 0.05) were identified, containing 3094 relatively upregulated genes and 1928 downregulated genes (Fig. 3a and Supplementary Table 2). We then calculated the adjusted *p* value of BP, KEGG and DO enrichment analyses. BP analysis displayed the most enrichment in positive regulation of kinase activity (Fig. 3b). In terms of KEGG pathways, the majority of the genes were enriched in the PI3K-Akt signaling pathway (Fig. 3c). DO analysis indicated that DEGs are mainly related to female reproductive organ cancer (Fig. 3d).

### Collection and Analysis of CR-DEGs

**Fig. 4:**
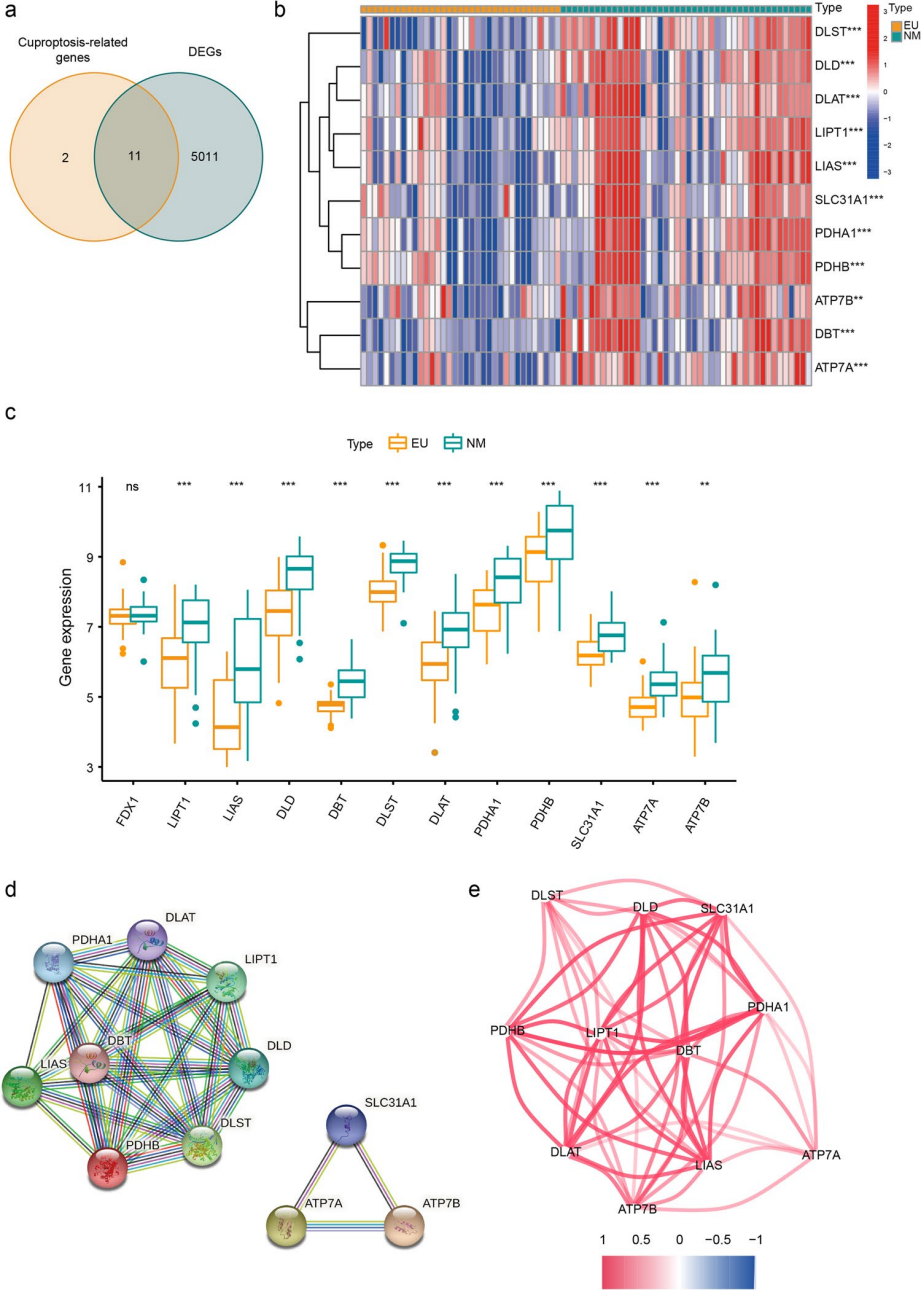
Identification and characteristics of CR-DEGs. **a** The Venn diagram showing the intersection of DEGs and cuproptosis-related genes. **b** The heat map of CR-DEGs. **c** The boxplot of CR-DEGs (***p < 0.001; **p < 0.01). **d** The PPI network of CR-DEGs. Plots represent genes, and the lines between each plot represent interaction between the proteins coded by these genes. **e** The co-expression network of CR-DEGs. Color scale represents the degree of expression levels’ association (red line, positive correlation; blue line, negative correlation)

In total, twelve cuproptosis-related genes were collected with reference to Tsvetkov et al.’s article [11]. Following this, we obtained eleven CR-DEGs from the intersection of cuproptosis-related genes and DEGs (Fig. 4a). The relevant information of CR-DEGs is displayed in Table 2. The heat map and boxplot of these CR-DEGs indicated that all CR-DEGs were relatively downregulated in EU samples than in NM samples (Fig. 4b c). As we can see from the PPI network, CR-DEGs can be divided into two groups according to their coded proteins’ functions and genes within the same group performed pretty close relationship with each other. One cluster included *PHDHA1*, *DLAT*, *LIPT1*, *DLD*, *DLST*, *PDHB*, *DBT* and *LIAS*. The other included *SLC31A1*, *ATP7A* and *ATP7B*, which work as copper-transporters (Fig. 4d). Moreover, the co-expression network also showed strong positive correlations above all CR-DEGs (Fig. 4e). The centrally regulatory genes were *PHDHA1*, *DLAT*, *LIPT1*, *DLD, PDHB*, *DBT*, *LIAS* and *SLC31A1*.

**Table 2:** Relevant information of CR-DEGs

Genes	Full name	Role in cuproptosis
*ATP7A*	ATPase copper transporting alpha	Copper exporter and intracellular distribution
*ATP7B*	ATPase copper transporting beta	Copper exporter and intracellular distribution
*SLC31A1*	Solute carrier fa	Copper importer
*LIAS*	Lipoic acid synthetase	Lipoylation process
*LIPT1*	Lipolytransferase 1	Lipoylation process
*DBT*	Dihydrolipoamide branched chain transacylase E2	A component of BCKDC, a direct target of lipoylation
*DLST*	Dihydrolipoamide S-succinyltransferase	A component of ɑ-KGDH, a direct target of lipoylation
*DLD*	Dihydrolipoamide dehydrogenase	A component of PDHC, ɑ-KGDH and BCKDC
*DLAT*	Dihydrolipoamide S-acetyltransferase	A component of PDHC, a direct target of lipoylation
*PDHA1*	Pyruvate dehydrogenase E1 subunit alpha 1	A component of PDHC
*PDHB*	Pyruvate dehydrogenase E1 subunit beta	A component of PDHC

BCKDC branched-chain ketoacid dehydrogenase complex, ɑ-KGDH alpha-ketoglutarate dehydrogenase complex, PDHC pyruvate dehydrogenase complex

### Identification of Characteristic CR-DEGs

**Fig. 5:**
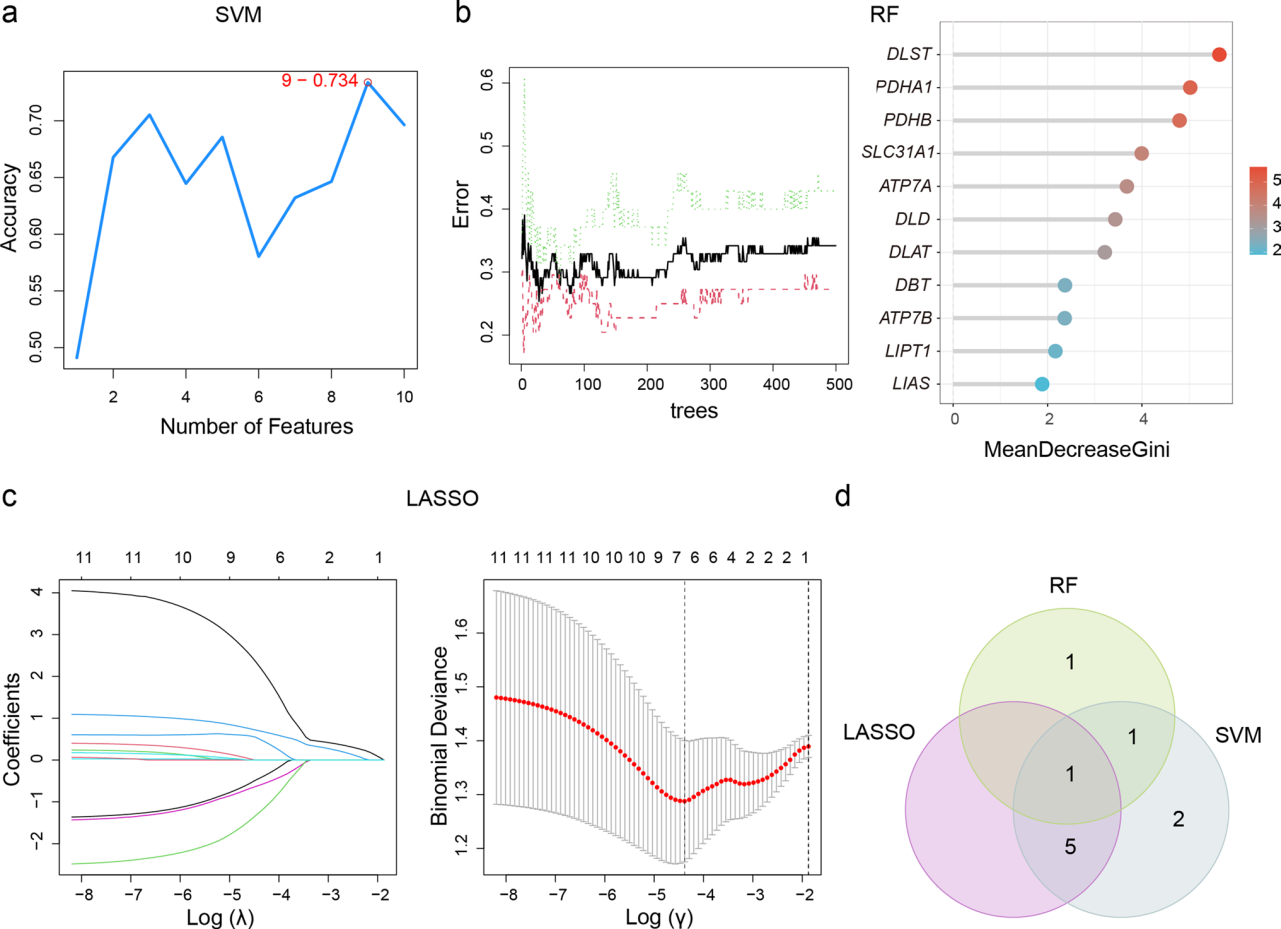
Identification of characteristic CR-DEGs. **a** Trends of related accuracy rate according to the number of features involved in the SVM model. **b** Trends of related errors according to the number of decision trees and mean decrease in Gini index of the RF model. **c** Lasso coefficient profiles and deviance profiles. **d** The intersection of SVM, RF and LASSO. SVM support vector machine, RF random forest algorithm, LASSO logistic regression with lasso regularization

According to Fig. 5a, the SVM algorithm obtained the highest accuracy when the top 9 genes (*PDHA1*, *SLC31A1*, *LIPT1*, *DLAT*, *ATP7A*, *DBT*, *DLD*, *FDX1*, *LIAS*) were included in the model. The average rank of each gene of SVM model is applied in Supplementary Table 3. The out-of-bag error rate became the lowest with 33 trees (Fig. 5b). According to the criterion of MDG score larger than 4, 3 genes (*DLST*, *PDHA1* and *PDHB*) were collected (Fig. 5b). Through LASSO analysis, we excluded the variables with zero coefficient and gained 6 genes (*LIPT1*, *DBT*, *DLAT*, *PDHA1*, *SLC31A1* and *ATP7A*) (Fig. 5c). The coefficients obtained from LASSO is shown in Supplementary Table 4. Eventually, Venn diagram revealed that *PDHA1* appeared in all three analyses (Fig. 5d).

### Construction and Quality Assessment of Risk-Scoring Model

**Fig. 6:**
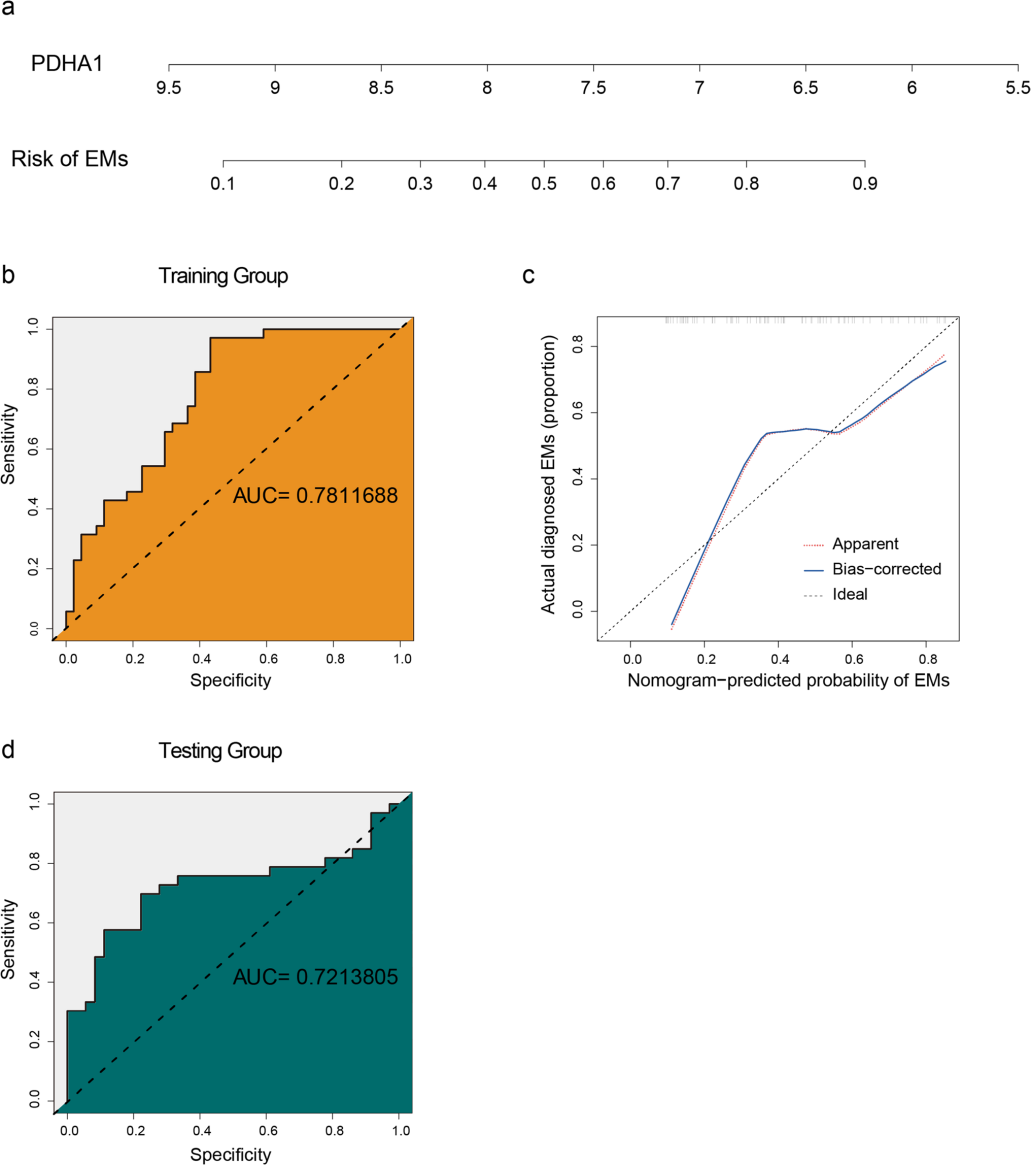
Construction and validation of developed model. **a** The risk-scoring nomogram of EMs. **b** The receiver operating characteristic curve (ROC) of the training group. **c** The calibration curve of nomogram-predicted probability of EMs. **d** The ROC of the testing group

To study the potential clinical value of *PDHA1*, we constructed a risk-scoring nomogram based on its expression level (Fig. 6a). The area under the ROC curve (AUC) of the training group was 0.781 (Fig. 6b). The calibration curve nearly coincided to ideal curve (Fig. 6d), and the *C*-index was 0.7. Additionally, the AUC and *C*-index of the testing dataset were 0.721 and 0.7, respectively. Both internal and external validations indicated the great performance of the developed model.

## Discussion

The high morbidity and complexity of EMs make it essential to explore the underlying mechanism leading to the unique PCD patterns of eutopic endometriotic cells. According to the latest study published in *Science*, cuproptosis is a novel type of PCD initiated by copper [11]. Our study represents the first attempt to explore the implication of cuproptosis in EMs.

To improve the quality of this study and avoid the potential influence of the menstrual cycle on gene expression levels [37], we only included endometrial tissues collected during the proliferative phase. DO analysis showed that EMs share a lot in common with reproductive organ cancer, which is consistent with previous literature, suggesting that cancer-associated mutations could be identified in EMs lesions [38]. The PI3K-Akt signaling pathway appeared most significant in the KEGG analysis, which was reported to regulate various mechanisms like ERβ and aromatase expression [39] and endometrial stromal cell migration [40] in EMs. Consistently, researchers found Akt inhibitor and mTOR inhibitor could inhibit the improvement of EMs both in vitro and in vivo [41, 42].

**Fig. 7:**
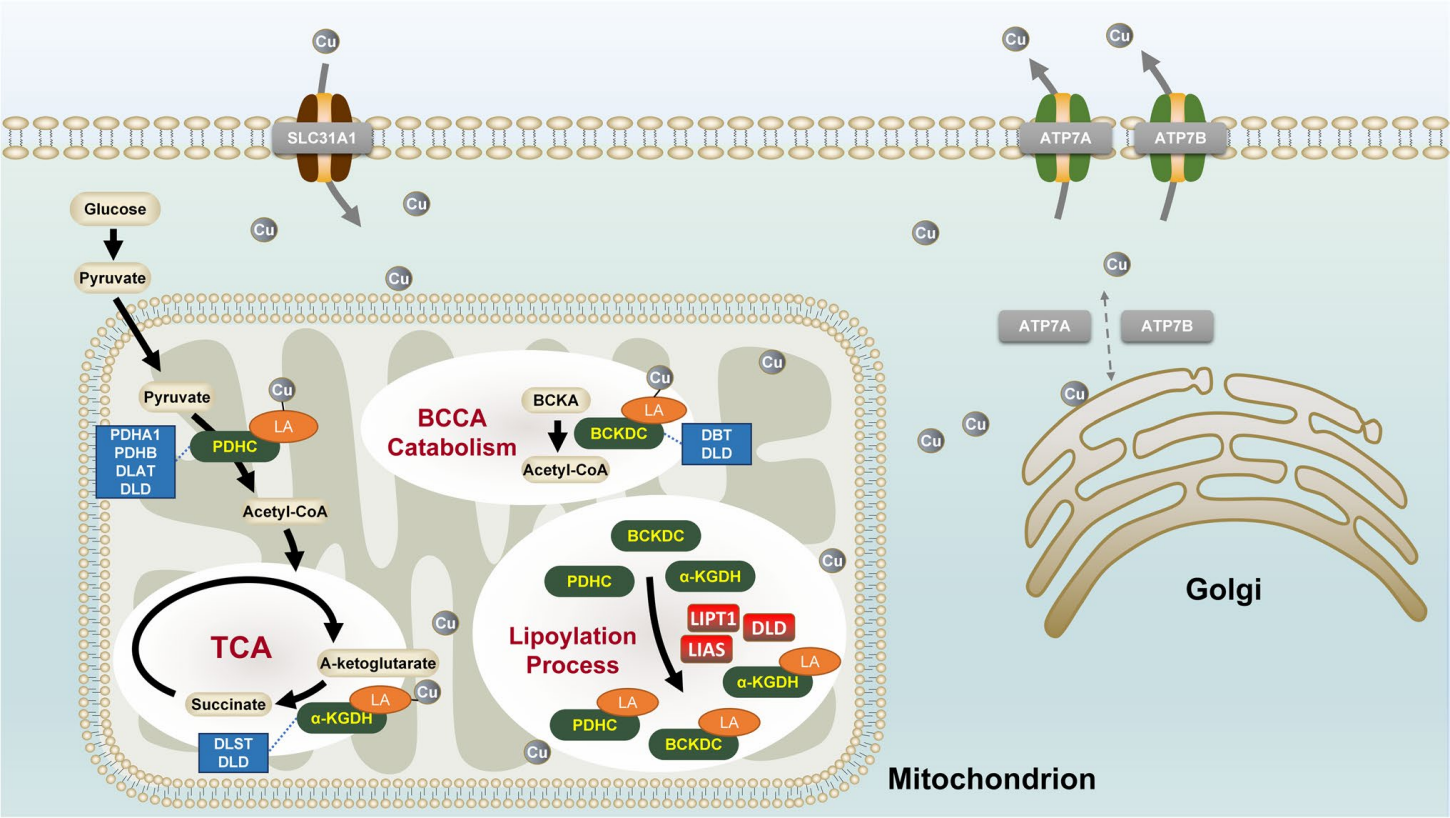
A brief overview of the roles of CR-DEGs in cuproptosis. Gray arrows, intracellular transport processes of copper ion; gray circles, copper ion; black arrows, abbreviated metabolic processes; green boxes, lipoyl-related enzyme complexes; blue boxes, important components of lipoyl-related enzyme complexes; red boxes, enzymes involving in lipoylation; orange circles, lipoyl group. BCAA branched-chain amino acids, BCKDC branched-chain ketoacid dehydrogenase complex, ɑ-KGDH alpha-ketoglutarate dehydrogenase complex, PDHC pyruvate dehydrogenase complex

According to the PPI network, eleven screened CR-DEGs could be divided into two groups. ATP7A, ATP7B and SLC31A1 work as copper transporters. ATP7A and ATP7B, cycling between the Golgi complex and the plasma membrane, take responsibility for copper intracellular distribution and excretion [43], while SLC31A1, located at the plasma membrane, behaves as the primary regulator of copper uptake [44]. Deficiency in *ATP7A* could cause Menkes disease, characterized by copper defect [45]. In line with our study, copper disorders in EMs have been well-reported in previous studies [12, 13]. As mentioned above, the target of cuproptosis is the lipoylation sites [46]. Lipoylation is a highly conserved posttranslational modification pattern that only occurs in four enzyme complexes, including branched-chain ketoacid dehydrogenase complex (BCKDC), alpha-ketoglutarate dehydrogenase complex (ɑ-KGDH), pyruvate dehydrogenase complex (PDHC) and glycine cleavage system (GCS). DLD, DLAT, PDHA1 and PDHB consist of PDHC, which connects glycolysis with the TCA cycle. DLST together with DLD constitutes a-KGDH, a crucial enzyme involved in the TCA cycle [47]. Besides, DBT and DLD are components of BCKDC, which acts as the rate-limiting enzyme in the branched-chain amino acid metabolism [48]. The activation of these three enzymes requires protein lipoylation, an ancient and highly conserved mechanism that relies on LIAS to convert the octanoyl moiety to lipoyl cofactor [48]. For better illustration, the brief overview of identified CR-DEGs is showed in Fig. 7.

All CR-DEGs were found to express significantly lower levels in the EMs group than in the healthy group, indicating that the endometrial tissues of EMs patients are insensitive to cuproptosis. Consistent with this finding, Siracusa et al. [49] found that EMs induced insensitivity towards multiple PCD via the Akt-mTOR pathway in a rat model. Likely, Ng et al. [10] proposed eutopic endometrial defects in EMs featured by resistance to ferroptosis, which allows refluxed endometrial debris to establish endometriotic lesions within the abdominal cavity. Therefore, we speculated that the relationship between the downregulation of cuproptosis level and the activation of PI3K-Akt-mTOR in EMs might also be involved in the underlying pathogenesis of EMs.

SVM, RF and lasso regularization were used to filter these CR-DEGs. *PDHA1* was speculated as the characteristic gene to construct a nomogram. Both internal and external tests indicated the great clinical value of *PDHA1* in EMs. PDHA1 is a major subunit of PDHC, the key rate-limiting enzyme that connects glycolysis with the TCA cycle. The inhibition and phosphorylation of *PDHA1* were mainly induced by pyruvate dehydrogenase kinases. Interestingly, positive regulation of kinase activity was the top enriched BP in our GO analysis. The suppression of PDHC would contribute to the Warburg effect, a mechanism characterized by a shift from oxidative phosphorylation towards glycolysis even with adequate oxygen supply [50]. Li et al. [51] reported that formaldehyde can induce ferroptosis via upregulation of the Warburg effect. Likewise, Icard et al. [52] reported that the Warburg effect causes modification of the TCA cycle and ROS production, which leads to resistance to multiple processes, including apoptosis and immune response. These studies indicated that this great alteration in mitochondrial metabolism could influence mitochondrial-dependent PCD and cause various diseases. Although little is known of the role of the Warburg effect in EMs, several research reported a lower expression level of *PDHA1* and a higher level of the Warburg effect in the untreated EMs group, as determined by western blot [53–55]. Therefore, it is reasonable to suspect that cuproptosis, the mitochondrial-dependent PCD, could be suppressed by upregulation of *PDHA1* and the Warburg effect it causes, which may explain the high proliferative and migratory potential of eutopic endometriotic cells in EMs. Besides, the Warburg effect was also known to be enhanced by the activated PI3K-Akt-mTOR pathway [56–58]. Collectively, we speculated that the interaction of *PDHA1* and the PI3K-Akt-mTOR pathway might involve in the Warburg effect and regulate the cuproptosis in EMs. Regretfully, the specific mechanism of this interaction remains unknown at present.

## Conclusion

In summary, *PDHA1*, the intersection of cuproptosis and the TCA cycle, was downregulated in eutopic endometrium of EMs patients. GO and KEGG analyses suggested that the kinase activity of PDHA1 and the PI3K-Akt-mTOR pathway may work in a tightly connected system and cause the Warburg effect, which might lead to insensitivity toward cuproptosis.

However, there are some flaws in our research. Firstly, analyses were not stratified according to each stage of rAFS due to the limitation of small sample sizes. Secondly, as it is a retrospective study obtained from public datasets, it lacks detailed clinical information, e.g. ages and treatment. Thirdly, the interrelation and specific mechanism of PDHA1 and the PI3K-Akt-mTOR pathway in EMs need further investigation.

## Supplementary Information

Below is the link to the electronic supplementary material.

Supplementary file1 (XLS 19 KB)

Supplementary file2 (XLS 373 KB)

Supplementary file3 (CSV 0 KB)

Supplementary file4 (XLS 0 KB)

## Data Availability

All data are available from the GEO database (https://www.ncbi.nlm.nih.gov/geo/).

## Code Availability

Codes generated or used during the study are available from the corresponding author by request.
